# An *Ex Vivo* Chicken Primary Bursal-cell Culture Model to Study Infectious Bursal Disease Virus Pathogenesis

**DOI:** 10.3791/58489

**Published:** 2018-10-04

**Authors:** Katherine L. Dulwich, Amin S. Asfor, Alice G. Gray, Venugopal Nair, Andrew J. Broadbent

**Affiliations:** ^1^The Pirbright Institute

**Keywords:** Immunology and Infection, Issue 140, Chicken, primary cells, bursa of Fabricius, B cells, virus, infectious bursal disease virus, IBDV

## Abstract

Infectious bursal disease virus (IBDV) is a birnavirus of economic importance to the poultry industry. The virus infects B cells, causing morbidity, mortality, and immunosuppression in infected birds. In this study, we describe the isolation of chicken primary bursal cells from the bursa of Fabricius, the culture and infection of the cells with IBDV, and the quantification of viral replication. The addition of chicken CD40 ligand significantly increased cell proliferation fourfold over six days of culture and significantly enhanced cell viability. Two strains of IBDV, a cell-culture adapted strain, D78, and a very virulent strain, UK661, replicated well in the *ex vivo* cell cultures. This model will be of use in determining how cells respond to IBDV infection and will permit a reduction in the number of infected birds used in IBDV pathogenesis studies. The model can also be expanded to include other viruses and could be applied to different species of birds.

**Figure:**
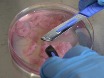


## Introduction

The global poultry industry is essential to secure enough food for an expanding human population. However, immunosuppression is the threat to food security and the welfare of affected birds and represents a key economic challenge to the industry. The majority of cases of immunosuppression in chickens are caused by the infection with immunosuppressive viruses, with those most responsible for impairing acquired immunity having a tropism for T and B lymphocytes^1^. In birds, the majority of B cells are located within an organ known as the bursa of Fabricius (BF). B cells are susceptible to infection with several immunosuppressive viruses, including those that cause lysis, such as IBDV and Marek's disease virus (MDV), and those that cause transformation, such as avian leukosis virus (ALV) and reticuloendotheliosis virus (REV).

In order to develop better strategies for controlling these infections, it is essential to characterize the interaction of the viruses with chicken B cells. However, when B cells are removed from a bird, they do not survive well in *ex vivo* culture^2^, making it difficult to perform a thorough analysis of the interactions of IBDV with chicken B cells, or the early events following ALV or REV infection. Consequently, many host cell-virus interactions have been studied *in vivo*^3^,^4^,^5^,^6^,^7^,^8^,^9^,^10^. Although these studies are informative, they involve the use of infected birds which suffer from diseases that can be severe.

The CD40 ligand is a molecule that induces B cell proliferation^11^. Following identification of the gene encoding chicken CD40L (chCD40L), a soluble fusion protein was engineered that, when added to the culture media, induced the proliferation of chicken primary B cells *ex vivo*^12^. In 2015, B cells cultured in this fashion were found to support the replication of MDV^2^ and in 2017, we and others found that primary bursal cells stimulated with chCD40L could be used as a model to study IBDV replication^13^,^14^. Here, we describe the isolation and culture of chicken primary bursal cells, the infection of the cells with IBDV, and the quantification of viral replication. Although we describe the method in the context of the BF, this could be applied to isolate and culture cells from different lymphoid organs.

## Protocol

All procedures with animals must be ethically approved in advance. In our institution, all procedures are performed in accordance with the UK Animal (Scientific Procedures) Act 1986 under Home Office Establishment, Personal and Project licenses, after the approval of the internal Animal Welfare and Ethical Review Board (AWERB).

### 1. Preparation of chCD40L

1. Culture HEK 293-msCD8-CD40L cells that stably express a soluble chCD40L construct in 1x Roswell Park Memorial Institute (RPMI) medium supplemented with 10% heat-inactivated (hi) fetal calf serum (FCS) and 1 µg/mL puromycin at 37 °C in an atmosphere containing 5% CO_2_. NOTE: These cells are available from The Pirbright Institute following the signing of the appropriate material transfer agreement.
2. Split the cells at a 1:5 density when confluent. Collect the supernatant (containing the soluble chCD40L construct) each time the cells are split, centrifuge it at 400 x *g* for 5 min to remove cellular debris, and store it at 4 °C.
3. When 500 mL of the supernatant has been collected, pool the liquid and filter-sterilize it through a 0.2 µm filter.
4. Concentrate the supernatant using centrifugal protein concentrators with a molecular-weight cutoff of 10 K according to the manufacturer’s instructions. Extract the concentrated supernatant from each column, pool it together, and filter-sterilize it by passing it through a 0.22 µm syringe filter.
5. Determine the final concentration to be used in experiments by serially diluting the chCD40L solution in 1x Iscove’s modified Dulbecco’s medium (IMDM) (described in step 2.4) and culturing primary bursal cells in the presence of the dilutions. Determine the number and percentage viability of the cells daily for up to a week. NOTE: The lowest concentration where cell proliferation and viability are adequate is the concentration to use in the assay. This is likely to be between 1:20 and 1:50.

### 2. Preparation of Solutions for Chicken Primary Bursal Cell Isolation

1. Prepare 1x Hanks’ balanced salt solution (HBBS) with calcium (Ca) by adding 10 mL of 10x HBBS with Ca to 90 mL of sterile H_2_O and 0.47 µL of 7.5% NaHCO_3_.
2. Prepare collagenase D stock solution at 8 mg/mL in 1x HBBS with Ca. Filter-sterilize the solution through a 0.2 µM filter. NOTE: It is advisable to prepare 5 mL aliquots and freeze them at -20 °C.
3. Prepare 1x RPMI medium supplemented with 5% hi FCS. Store the media at 4 °C.
4. Prepare 1x 500 mL of IMDM supplemented with 8% hi FCS, 2% hi chicken serum, 50 mM β-mercaptoethanol, 50 µL of insulin-transferrin-sodium-selenite, and 1% penicillin/streptomycin. Store the media at 4 °C. NOTE: Prepare all the above-mentioned solutions in advance.
5. Prepare 1x HBBS with Ca. Store the solution on ice.
6. Prepare 1x HBBS without Ca by adding 10 mL of 10x HBBS without Ca to 90 mL of sterile H_2_O, 0.47 µL of 7.5% NaHCO_3_, and EDTA at a final concentration of 10 mM. Store the solution on ice.
7. Prepare 1x collagenase D solution by adding 5 mL of collagenase D stock solution to 13 mL of HBBS with Ca to make a total of 18 mL. Store the solution on ice. NOTE: Prepare the solutions mentioned in steps 2.5–2.7 on the day of the experiment.

### 3. Removal of the Bursa of Fabricius (BF)

1. Rear and hatch chickens in an appropriate, approved facility and humanely cull them at 2–3 weeks of age. NOTE: Use institute-approved methods for culling.
2. **Collect the BF from each chicken using aseptic techniques.** NOTE: Use the protocols in place at the institution.
  1. Place the carcass in dorsal recumbency and sterilize the skin and feathers overlaying the abdomen and thorax with a solution of 70% ethanol, diluted in water.
  2. Make a ventral midline incision in the lower abdomen using a sterilized scalpel or scissors.
  3. Locate the bursa of Fabricius, which is connected to the caudal section of the colon, cranial to the cloaca.
  4. Using sterilized forceps and scissors, cut the bursa of Fabricius free from the colon. Take care to avoid puncturing the gut.
3. Place the organ in cold PBS and transfer it to the laboratory on ice. NOTE: Primary cells should be isolated as soon as possible after the organ harvest.

### 4. Isolation of Chicken Primary Bursal Cells

1. Working in a microbiological safety cabinet, wash the BF at least 3x in 30 mL of cold PBS.
2. Transfer the tissue to a Petri dish (92 mm in diameter, 21 mm in height) and add 5 mL of 1x collagenase D solution.
3. Using sterile scissors or a scalpel blade, cut the BF into pieces of less than 5 mm in diameter.
4. Incubate the tissue at 37 °C with periodic gentle agitation for 30 min. NOTE: The collagenase solution will begin to digest the tissue. 
5. Using a sterile Pasteur pipette, repeatedly aspirate the mixture to encourage disintegration of the tissue. If necessary, cut the tissue into smaller pieces.
6. Add another 5 mL of 1x collagenase D solution to the tissue and incubate it at 37 °C with periodic gentle agitation for another 30 min.
7. Repeat steps 4.6 and 4.7 until the tissue is completely digested. NOTE: There will be small granules that do not dissolve further.
8. Pass the cell suspension through a 100 µm cell strainer into 20 mL of 1x HBBS without Ca.
9. Centrifuge the cell suspension at 400 x *g* for 5 min.
10. Discard the supernatant and resuspend the pellet in 10 mL of 1x RPMI with 5% FCS.
11. Either overlay 10 mL of the cell suspension on top of 5 mL of density gradient media containing polysucrose and sodium diatrizoate or underlay 5 mL of density gradient media beneath the 10 mL cell suspension. Ensure there is a clear interface between the two.
12. Centrifuge the overlay at 900 x *g* for 20 min at 4 °C. Lower or remove the centrifuge break. NOTE: The cells should form a band at the interface between the cell media and the density gradient media.
13. Using a sterile Pasteur pipette, remove the cells and place them in cold PBS. Wash the cells 3x by centrifuging them at 400 x *g* for 5 min and resuspending them in cold PBS.

### 5. Culture of Chicken Primary Bursal Cells

1. Centrifuge the cell suspension at 400 x *g* for 5 min and resuspend them in 1x complete IMDM.
2. Take an aliquot of the cell suspension, add it to a Trypan blue solution and count the number of viable cells that exclude the Trypan blue. Determine the number of cells and the percentage viability.
3. Centrifuge the cell suspension at 400 x *g* for 5 min and resuspend it in complete IMDM supplemented with a 1:20 dilution of chicken CD40L at a density of 1 x 10^7^ cells/mL. Titrate the concentrated supernatant containing the chCD40L to determine the optimal dilution, which is likely to lie in the range of 1:10 to 1:50.
4. Culture the cells in either 96- or 24-well plates at 37 °C for 48–72 h. U-bottomed 96-well plates are preferable to flat-bottomed plates. NOTE: Cells can also be cultured at 40 °C

### 6. Infection of Chicken Primary Bursal Cells with IBDV

1. 48–72 h post-isolation, thaw an aliquot of the virus, vortex the sample, and store it on ice.
2. Resuspend the primary bursal cells, take a 10-µL aliquot of the cell suspension, add it to 10 µL of a Trypan blue solution, and determine the number of cells and percentage viability.
3. Dilute the virus in 1x complete IMDM to the appropriate multiplicity of infection (MOI) to make the virus inoculum and vortex.
4. Centrifuge the cell suspension at 400 x *g* for 5 min.
5. Remove the supernatant and resuspend the cells in the virus inoculum.
6. Incubate the cell suspension at 37 °C for 1 h with periodic agitation.
7. Centrifuge the cell suspension at 400 x *g* for 5 min, remove the virus inoculum, and wash the cells in 1x complete IMDM media.
8. Centrifuge the cell suspension at 400 x *g* for 5 min, remove the supernatant, and resuspend the cells in complete IMDM media supplemented with chicken CD40L at a density of 1 x 10^7^ cells per mL.
9. Culture the cells in either 96- or 24-well plates at 37 °C. NOTE: Cells can also be cultured at 40 °C

### 7. Quantification of IBDV Replication in Chicken Primary Bursal Cells

1. At the desired time-point postinfection, resuspend the cells, transfer them to an appropriate tube, centrifuge them at 400 x *g* for 5 min and harvest the supernatant for virus titration by either plaque assay or TCID_50_ assay as per the Reed-Muench method^15^.
2. Wash the cells in 1 mL of PBS and prepare them either for immunostaining with an antibody specific to IBDV, or extract RNA using an appropriate kit (following the manufacturer’s instructions) and perform reverse transcription quantitative polymerase chain reaction (RT-qPCR) using primers specific for an IBDV gene (Forward, GAGGTGGCCGACCTCAACT; Reverse, GCCCGGATTATGTCTTTGAAG). Mock-infected cell cultures should be used as a control.

## Representative Results

### Chicken Primary Bursal Cells Can Be Cultured in the Presence of Chicken CD40L

When chicken primary bursal cells were cultured in the presence of soluble chCD40L, the number of cells increased fourfold from 9.02 x 10^5^ to 3.63 x 10^6^ per mL over a period of 6 days, in contrast to when it was absent (*p* <0.05) (Figure 1A). Cell viability was also significantly improved, for example from 25% at day 3 post-culture in the absence of chCD40L to 48% in the presence of chCD40L (*p* <0.05) (Figure 1B)^13^.

### Chicken Primary Bursal Cells Can Support the Replication of Both Cell-culture Adapted and Very Virulent Strains of IBDV

Mock-infected and infected cell cultures were fixed 18 h postinfection, labeled with a monoclonal antibody against IBDV VP2 and a secondary antibody conjugated to Alexa Fluor 488, and counterstained with DAPI. Infected cells had evidence of green fluorescence around the nucleus (Figure 2A), consistent with the presence of IBDV in the cytoplasm. This was evident for two strains of IBDV, a cell-culture adapted strain, D78, and a very virulent strain, UK661 (Figure 2A). At 5, 18, 24, and 48 h postinfection, RNA was extracted from infected cultures and subjected to RT-qPCR with primers specific to a conserved region of the IBDV VP4 gene. The expression of VP4 was first normalized to the house-keeping gene TBP and then expressed as fold change relative to mock samples in a ΔΔCt analysis. IBDV VP4 expression increased to 16,603 copies at 48 h postinfection with D78 and 38,632 copies at 48 h postinfection with UK661. Taken together, these data demonstrate that the chicken primary bursal cells could support the replication of cell-culture-adapted and very virulent IBDV strains^13^.

**Figure:**
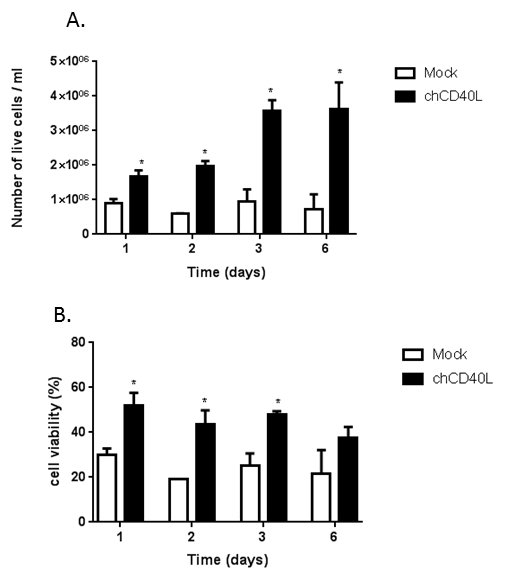

**Figure 1: Chicken primary bursal cells can be cultured in the presence of chicken CD40L.** Chicken primary bursal cells were cultured in the presence or absence of chCD40L (black bars and white bars, respectively). (**A**) The number of live cells and (**B**) the percentage of viable cells were determined at the indicated time-points postinfection. The data shown are representative of at least three replicate experiments, the error bars represent the standard deviation of the mean, and the statistical significance was determined using a paired Student's *t*-test at each time-point, * *p* <0.05. This figure has been modified with permission from Dulwich *et al.*^13^.

**Figure:**
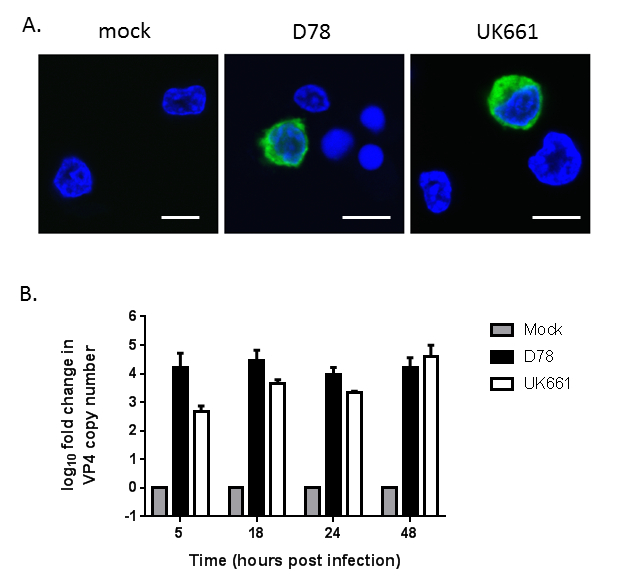

**Figure 2: Chicken primary bursal cells can support the replication of both cell-culture adapted and very virulent strains of IBDV.** (**A**) Chicken primary bursal cells were mock-infected or infected with either D78 or UK661 and a sample from each culture was fixed, labeled and imaged: IBDV VP2, green; nuclei, blue. Scale bar = 7 µm. (**B**) RNA was extracted at the indicated time-points postinfection, reverse-transcribed, and a conserved region of the IBDV VP4 gene was amplified by quantitative PCR. The log_10_ fold change in VP4 copy number was normalized to the TBP housekeeping gene and expressed relative to mock-infected samples as per the 2^-^^ΔΔCT^ method. The data shown are representative of at least three replicate experiments, and the error bars represent the standard deviation of the mean. This figure has been modified with permission from Dulwich* et al.*^13^.

## Discussion

In this study, we describe the successful culture of chicken primary bursal cells *ex vivo* in the presence of soluble chCD40L and demonstrate that these cells can support the replication of an attenuated strain and a very virulent strain of IBDV. This *ex vivo* model can be used to determine how the cells respond to an IBDV infection^13^, which has distinct advantages over *in vivo* and *in vitro *studies.

When harvesting the BF, it is critical to not puncture the gut so as to avoid bacterial contamination of the isolated bursal cells. In addition, it is important to isolate the primary cells as soon as possible after the organ harvest to limit cell death. The need to use chCD40L is a limitation of the technique; however, work conducted by Soubies* et al.* shows that the use of phorbol 12-myristate 13-acetate (PMA) to prolong bursal cell viability instead of chCD40L^14^ may enable the model to be adopted by a greater number of laboratories. The protocol outlined above determines the optimal concentration of chCD40L empirically, by culturing primary B cells in serially diluted concentrations of the molecule and observing cell proliferation and viability. One potential modification to the protocol could be to purify the chCD40L molecule and to add a specific concentration to the cell culture media to avoid batch-to-batch variability.

*In vivo* studies have shown that following IBDV infection, there is an increase in the expression of genes involved in pro-inflammatory cytokine responses, Type I IFN responses, and apoptosis in the BF^5^,^9^,^10^. However, following infection, there is an influx of inflammatory cells and effector T cells into the BF, which differ in the genes they express compared to the infected B cell population^9^. It is, therefore, difficult to interpret how infected cells respond to IBDV. To address this, some research groups have characterized the transcriptional response of cells infected with IBDV in culture^16^,^17^,^18^,^19^,^20^. These *in vitro* studies have the advantage of well-defined MOIs and time-points postinfection. However, *in vitro* studies have typically been characterized in either fibroblast cells^16^,^17^,^20^ or dendritic cells^18^. While providing some insight into host cell-IBDV interactions, the current belief is that the infection of B cells is crucial to the pathogenesis of IBDV and, therefore, the relevance of the data cannot be overinterpreted. Prior to our *ex vivo* bursal cell culture model, only one study had characterized the cellular response of B cells to IBDV infection^19^; however, this study utilized an immortalized B cell line that was transformed due to infection with ALV, limiting the conclusions that could be made. In contrast, the *ex vivo *model of IBDV infection described here allows researchers to retain the advantages of *in vitro* studies, such as defined MOIs and time-points, while studying the interactions of the virus with its relevant host cell. As the primary bursal cells are obtained from uninfected BF tissue, there are no inflammatory or T cells present, and we have demonstrated by flow cytometry (using standard conditions) that, following chCD40L stimulation, 97% of the cell population is positive for the B cell marker Bu-1 (data not shown). Given that 3% of the cells are Bu-1 negative, it will be interesting to determine whether these cells become infected with IBDV and explore their gene expression and contribution to the pathogenesis.

We anticipate that the *ex vivo* chicken primary bursal cell culture model can also be expanded to study the host cell-virus interactions of other B-cell tropic viruses infecting chickens, such as ALV or REV, and could also be expanded to other avian species (*e.g.*, ducks or turkeys). The ability to culture primary bursal cells *ex vivo* also opens up the possibility to study aspects of the pathogenesis and immunosuppression caused by these viruses without the need to infect birds. As *in vivo* studies cause significant morbidity, this will have a substantial impact on the replacement, refinement, and reduction of the use of animals in research.

In summary, the *ex vivo *chicken primary bursal cell culture model described here has the potential to expand the understanding of how avian B-cell tropic viruses interact with their host cells while reducing the number of birds used in *in vivo* infection studies. The techniques can be applied to multiple lymphoid organs, multiple viruses, and, potentially, multiple species of birds, making it an attractive model that can contribute to the avian virology and immunology fields.

## Disclosures

The authors have nothing to disclose.
